# Single breath-hold real-time MR cardiac cine for evaluation of left ventricular function

**DOI:** 10.1186/1532-429X-17-S1-O63

**Published:** 2015-02-03

**Authors:** Tomoyuki Kido, Kouki Watanabe, Yuta Urusibata, Masashi Nakamura, Michaela Schmidt, Michael O Zenge, Teruhito Mochizuki

**Affiliations:** 1 Radiology, Ehime University, Toon, Japan; 2 Saiseikai Matsuyama Hospital, Matsuyama, Japan; 3 Siemens Japan K.K., Research & Collaboration Department, Shinagawa, Japan; 4 Siemens AG, Erlangen, Germany

## Background

Magnetic Resonance (MR) cardiac cine is generally accepted as the gold standard for left ventricular (LV) volume assessment. Recently, Real-Time (RT) cine with sparse sampling technique and iterative reconstruction has been applied to accelerate cine MR. However, in prospective electrocardiogram (ECG) triggered RT cine MR, it may be difficult to capture the true end diastole phase because of the finite time needed to detect the next ECG trigger, which may lead to underestimation of end diastolic volume (EDV), stroke volume (SV), and ejection fraction (EF) when compared with retrospective ECG gated standard cine MR.

In this study we propose an alternative approach to overcome this limitation, by acquiring sparse RT cine MR data over two heart beats, to capture the complete end diastole between the first and second heart beats. The purpose of this study was to evaluate the diagnostic quality and accuracy of RT-based single-breath-hold cine MR for the quantification of LV function compared with standard multi-breath-hold cine MR.

## Methods

Twenty participants (10 volunteers: mean age 27±4 years; 10 patients: mean age 69±8 years with different LV pathologies) underwent both RT cine (temporal/spatial resolution: 41 ms/1.7×1.7×6 mm^3^; acceleration factor 12.8) using a prototype sequence with sparse sampling and iterative reconstruction (Liu J et al. ISMRM; 2012) and standard segmented cine MR (temporal/spatial resolution 41 ms/1.7×1.7×6 mm^3^; acc. factor 3) for the assessment of LV function on a 3 Tesla MRI scanner (Magnetom Skyra, Siemens AG). The cine images were obtained in a stack of 8 contiguous short axis slices spanning the entire LV from base to apex. The image quality was scored (1 = nondiagnostic; 2 = poor; 3 = adequate; 4 = good; 5 = excellent). Results in terms of EF, EDV, end systolic volume (ESV), SV, and LV mass for sparse RT cine and standard cine MR were compared with the Wilcoxon signed-rank test, Pearson's correlation coefficient (r), and Bland-Altman analysis. In addition, assessment of intra-observer and inter-observer variation were performed.

## Results

Compared with standard, the RT cine yielded slightly worse image quality scores (4.9±0.4 for standard vs. 4.5±0.7 for RT; P < 0.05). However, for RT cine, all scores were above 3 (adequate), suggesting that acceptable diagnostic image quality can be achieved. Standard and RT cine showed good agreement: EF (59.1±8.6% vs. 58.1±8.6%; r=0.93; p=0.14); EDV (133.7±44.1 ml vs. 132.5±43.9 ml; r=0.98; p=0.75); ESV (56.7±29.7 ml vs. 57.3±29.2 ml; r=0.98; p=0.19); SV (76.9±19.4 ml vs. 75.2±20.1 ml; r=0.94; p=0.36); LV mass (84.7±34.6 ml vs. 82.7±36.3 ml; r=0.98; p=0.30), respectively. The intra-observer and inter-observer agreement for all parameters was good (slopes 0.86 to 1.09; r=0.97 to 0.99).

## Conclusions

The results demonstrated that sparse RT cine MR evaluates LV function and volumes with excellent accuracy. The single-breath-hold sparse RT cine MR has the potential to replace the multi-breath-hold standard cine MR.

## Funding

N/A.

**Table 1:** Comparison of Standard Cine MR Versus RT Cine MR

	Standard cine	RT cine	Mean Difference	p Value
LVEF (%)	59.1 ± 8.6	58.1 ± 8.6	1.4 ± 3.5	0.14

LVEDV (ml)	133.7 ± 44.1	132.5 ± 43.9	1.1 ± 10.3	0.75

LVESV (ml)	56.7 ± 29.7	57.3 ± 29.2	-1.0 ± 6.3	0.19

LVSV (ml)	76.9 ± 19.4	75.2 ± 20.1	2.0 ± 7.5	0.36

LV mass (g)	84.7 ± 34.6	82.7 ± 36.3	1.1 ± 10.4	0.30

**Figure 1:**
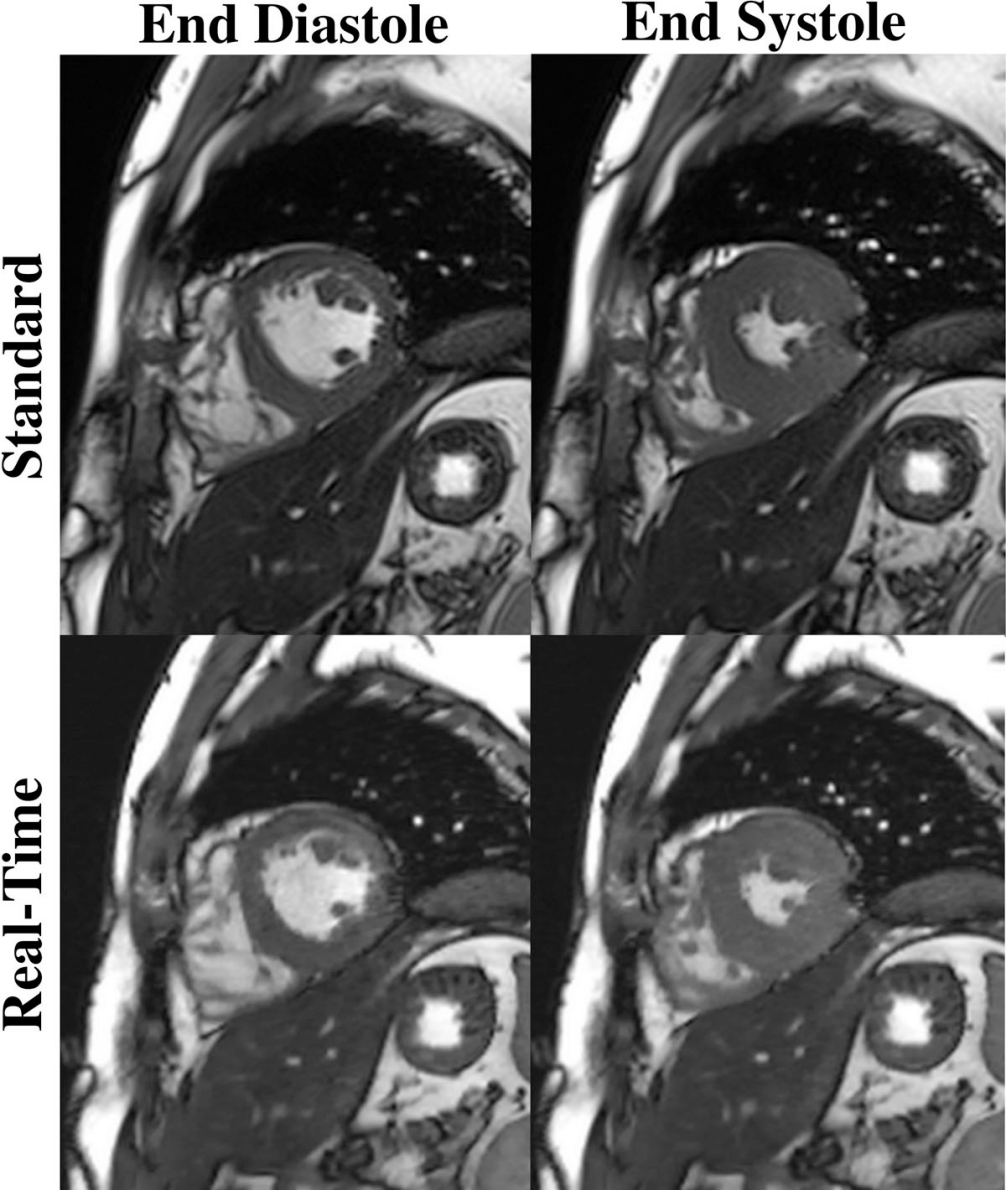
Image example comparing the standard cine versus the sparse RT cine

